# Circularised 1 and 2 LTR DNA circles are present in freshly- and chronically-infected cell lines and patient PBMCs, indicating ongoing reverse transcriptase usage

**DOI:** 10.1186/1742-4690-8-S1-A51

**Published:** 2011-06-06

**Authors:** James M Fox, Silva Hiburn, Lucy B M Cook, Maria Antonietta Demontis, Aileen Rowan, Fabiola Martin, Graham P Taylor

**Affiliations:** 1 Centre for Immunology and Infection, Hull York Medical School and Department of Biology, University of York, York, UK; 2 Section of Infectious Diseases, Imperial College, London, W2 1PG, UK; 3 Department of Immunology, Imperial College, London, W2 1PG, UK

## Background

After cell entry, HTLV-1 RNA is reverse transcribed and integrated into the host genome using its own reverse transcriptase (RT) and integrase (IN) enzymes. However, some unintegrated DNA circularises into 1 or 2 LTR DNA. Little is known about these unintegrated HTLV-1 DNA circles. Similar to HIV an inhibition of RT should decrease, and an inhibition of IN increase the 1/2 LTR DNA levels.

## Questions

Can 1/2 LTRs be detected in chronically infected MT2 cells and patient samples? Can 1/2 LTRs be detected in freshly infected CEM cells? Are 1/2 LTRs a marker of RT and IN activity? Can raltegravir (RGV), an IN inhibitor, prevent fresh infection?

## Methods

Detection of 1/2 LTRs in MT2 cells and patient PBMCs (4 ATLL, 4 HAM/TSP, 4 AC) using nested PCRs. Co-culture of gamma-irradiated MT2 with uninfected CEM cells with and without 1µM RGV for 2 weeks. DNA was extracted from 1x10^6^ cells at days 3,7,10 and 14 for proviral load and 1/2 LTR DNA detection and quantification.

## Results

1/2 LTRs were detected in MT2 (1LTR: 1 copy/600; 2LTR: 1copy/2000 cells); in all 16 patient PBMCs; in 3hr and day 14 infected CEM cells. Data on 1/ 2 LTR quantification in all patients and RGV inhibition study will be available for presentation.

## Conclusion

Both 1 and 2 LTRs are detected in freshly (CEM) and chronically infected cells (MT2, patient PBMCs), indicating ongoing usage of RT. 2 LTR DNA circles are detected at significantly lower levels than 1 LTRs in MT2 cells.

